# Predictive biomarkers of response to bacillus Calmette‐Guérin immunotherapy and bacillus Calmette‐Guérin failure for non‐muscle invasive bladder cancer

**DOI:** 10.1111/iju.14921

**Published:** 2022-05-22

**Authors:** Ziting Wang, Wei Zheng So, Kep Yong Loh, Yew Koon Lim, Ratha Mahendran, Qing Hui Wu, Edmund Chiong

**Affiliations:** ^1^ Department of Urology National University Hospital Singapore; ^2^ Department of Internal Medicine Singapore General Hospital Singapore

**Keywords:** bladder cancer, immunotherapy, recurrence

## Abstract

Within the heterogeneous population of patients with bacillus Calmette‐Guérin failure, there are clear differences in prognosis and therapy with regard to the timeline when bacillus Calmette‐Guérin failure occurred. There are a variety of classifications which include bacillus Calmette‐Guérin refractory disease, relapsing, unresponsive, and intolerant. Further profiling of these patients may help to shed light on other forms of therapy that are less radical. We hereby summarize the different biomarkers that predicts for response to bacillus Calmette‐Guérin immunotherapy and bacillus Calmette‐Guérin failure for non‐muscle invasive bladder cancer.

**Abbreviations & Acronyms**
AUC: area under the curve
BCG: bacillus Calmette‐Guérin
CIS: carcinoma in situ
CNV: copy number variant
CUETO: Club Urológico Español de Tratamiento Oncológico
EAU: European Association of Urology
EORTC: European Organisation for Research and Treatment of Cancer
FISH: fluorescence in situ hybridization
HR: hazard ratio
IL: interleukin
IQR: interquartile range
LMR: lymphocyte‐to‐monocyte ratio
NAL: neoantigen load
NLR: neutrophil‐to‐lymphocyte ratio
NMIBC: non‐muscle invasive bladder cancer
OR: odds ratio
PD‐L1: programmed death‐ligand 1
PLR: platelet‐to‐lymphocyte ratio
SNP: single‐nucleotide polymorphism
TMB: tumor mutation burden
TME: tumor microenvironment
TSG: tumor suppressor gene
TURBT: transurethral resection of bladder tumor

## Introduction

The definition of BCG failure has evolved over the years. The most recent definition in the EAU guidelines broadly defined BCG failure as any high‐grade disease occurring during or after BCG therapy.^1^ Prior to labeling the patient as BCG failure, the exclusion of disease in the upper urinary tracts and prostate should be established, along with ensuring that the patient has had adequate BCG, which is defined as at least five of six doses of an initial induction course plus at least two of six doses of a second induction course or two of three doses of maintenance therapy.^2^ Within this heterogeneous population of patients with BCG failure, there are clear differences in prognosis and therapy with regard to the timeline when BCG failure occurred. There are a variety of classifications which include BCG refractory disease, relapsing, unresponsive, and intolerant.^3^ The next line of treatment is a cystectomy, which some may deem as overtreatment for a non‐muscle invasive disease. Further profiling of these patients may help to shed light on other forms of therapy that are less radical. We hereby summarize the different biomarkers that predicts for response to BCG immunotherapy and BCG failure for NMIBC.

## Predictors of BCG response and failure

### Clinical‐pathological scoring systems

When BCG therapy was initially officialized as a therapeutic option, clinical parameters were highly anticipated to aid in deciding BCG provision for bladder cancer patients. Various clinical trials and numerous observational studies were en route to report their preliminary findings and declare significant predictors of poor oncological outcomes. Of note was the EORTC, as well as the CUETO groups. First introduced in the 2008 EAU Guidelines on TaT1 bladder cancer,^4^ the EORTC scoring system is based on a randomized cohort analysis of 2596 patients diagnosed with non‐muscle invasive tumors,^5^ developed primarily to facilitate management options post‐TURBT. This validated risk score has been consistently recommended by the EAU Guidelines for use in routine clinical practice over the last decade,^6^, ^7^, ^8^, ^9^ reaffirming its reliability in determining recurrence and progression risk at 1 and 5 years posttreatment. However, an important underlying drawback exists particular to this model – patients were mainly managed with intravesical chemotherapy. This inadvertently undermines the accuracy of predicting BCG failure when applied to a cohort that is treated with BCG, the current gold standard for treatment.

To circumvent this incongruity, the CUETO framework was tailored specifically as a scoring model for patients treated with 12 doses of intravesical BCG therapy over a 5‐ to 6‐month period post‐TURBT.^10^ Likewise, it aims to ascertain the risk of recurrence and progression of cancer by assessing seven significant factors: gender, age, prior recurrence status, number of tumors, T category, associated CIS, and the World Health Organization 1973 tumor grade. Patients are then categorized into four risk groups as governed by the aforementioned risk factors, with the risks of recurrence and progression clearly delineated for each group.

Indeed, while the CUETO model has been aligned to focus on the BCG‐treated population, patients were not standardized to undergo the standard treatment protocol of 1–3 years of maintenance therapy, as defined by the Southwest Oncology Group.^3^ Rather, they received six cycles of two‐weekly maintenance treatment across a 5‐ to 6‐month period, with the maximum period attained to be of a year's duration. Notably, this will naturally deviate from predicting a trustable response in high‐risk patients on a 3‐year BCG maintenance regime, which is in fact the current recommended treatment.^11^ Moreover, the CUETO model was unexpectedly seen to overestimate the risk of recurrence and likelihood of disease progression in high‐risk patients in several cohort studies.^12^, ^13^, ^14^

In more recent times, the EAU 2021 Guidelines have reclassified patients into four risk groups, with the additional clinical risk factors of age, multiple papillary tumors, and tumor diameter. Admittedly, all these models do appear to be sufficiently consistent and reproducible. However, aside from the limitations as mentioned above, the selected factors are still unable to account for the wide confidence intervals for recurrence and progression. Based on the new EAU risk groups, a patient within the very high‐risk group was cited as having a 53% 10‐year progression risk, with confidence intervals that range from 36 to 73%.^1^ This suggests that there are significant factors in play that are not captured within the risk stratifications.

### Clinical characteristics

Gender and age have been identified as potential markers of responsiveness or failure to BCG therapy. In particular, the female gender was previously validated as a significant poor prognostic indicator of NMIBC recurrence in patients who were enrolled within the CUETO model.^13^, ^15^ It is hypothesized that the pathophysiology involves the association of bladder carcinogenesis with female hormonal receptors.^16^, ^17^ Other hypothesized mechanisms include the gender‐varying immunomodulatory activity which was seen within pathologic samples of NMIBC patients. Within this Japanese cohort, females demonstrated increased regulatory T‐cell counts within the TME, of which elevated levels independently predicted disease recurrence.^18^

Advanced patient age has also been postulated as a poor prognostic factor of BCG responsiveness in NMIBC. A study of 805 patients with bladder CIS who were treated with BCG therapy showed that at 5 years, the recurrence rate was significantly higher in the age group more than 70 years old (73% *vs* 63%; *P* = 0.005).^19^ In the EORTC trial 30 911, it was reported that patients above the age of 70 years not only demonstrated poorer long‐term prognosis with regard to BCG responsiveness, but also demonstrated worse progression‐free survival, overall survival, and NMIBC‐specific survival.^20^ It is hypothesized that this is due to the age‐related attenuation of the immune response, diminishing the therapeutic effects of BCG treatment.^21^

### Biochemical immune response markers

Methods to objectively quantify the extent of immune cell response after the induction of BCG therapy have been purported to herald optimal outcomes of treatment,^22^ as seen in Table 1. Hematological biomarkers include NLR, PLR, and LMR.^23^, ^24^, ^25^, ^26^ In a retrospective cohort study involving 100 high‐risk NMIBC patients, higher mean NLR values were seen in the BCG non‐responder group (3.65 ± 1.16 *vs* 2.61 ± 0.77; *P* = 0.01). Moreover, increase in NLR correlated significantly with disease recurrence and progression risk scores.^27^ In a similar vein, another study also concluded that an optimal cutoff of NLR >2.5 served as an independent predictor of disease recurrence and prognosticated worse recurrence‐free survival in NMIBC patients, particularly within the subgroup treated with BCG therapy.^28^ A meta‐analysis of 15 studies comprising 5354 patients reported that elevated PLR exhibited poorer progression‐free survival and disease‐free survival.^29^ Although not limited to BCG therapy, it is also interesting to note that high PLR correlated significantly with patient age >65 years old. This finding postulates that bladder cancer prognosis might be confounded by either that of an elevated PLR or advanced age. Likewise, albeit not within the population of patients receiving BCG therapy, LMR was reported to be a potential prognostic marker for survival in bladder cancer patients who underwent radical cystectomy.^30^

**Table 1:** Biochemical markers associated with the outcomes of intravesical BCG therapy in NMIBC

Predictors	Author	Number of patients/samples	Outcome				
			Recurrence (*P*‐value/HR/OR/correlation coefficient [*R*])	Recurrence‐free survival (*P*‐value/HR/OR/correlation coefficient [*R*])	Progression (*P*‐value/HR/OR/correlation coefficient [*R*])	Progression‐free survival (*P*‐value/HR/OR/correlation coefficient [*R*])	Response (*P*‐value/HR/OR/correlation coefficient [*R*])
NLR	Getzler *et al*.^28^	113	HR 3.7 (95% CI 1.2–11.9, *P* = 0.023)	Lower RFS (21.3 *vs* 34.1 months in NLR ≥2.5 and <2.5, respectively, *P* = 0.013)			
	Racioppi *et al*.^27^	100	NLR value correlated significantly with recurrence risk score (*R* = 0.55, *P* = 0.01)		NLR value correlated significantly with progression risk score (*R* = 0.49, *P* = 0.01)		
LMR	Adamkiewicz *et al*.^23^	125			AUC 3.25, OR 0.54, *P* < 0.001		
FISH	Kamat *et al*.^31^	126	Positive FISH result *vs* negative FISH result: at baseline (38.3% *vs* 17.8%, *P* = 0.020), at 6 weeks after BCG initiation (34.0% *vs* 13.5% *P* = 0.008), at 3 months after BCG (58.3% *vs* 15.3%, *P* < 0.001), and at 6 months after BCG (69.2% *vs* 16.9%, *P* < 0.001)		Positive FISH result *vs* negative FISH result: at baseline (19.8% *vs* 4.4%, *P* < 0.032), at 6 weeks (*P* = 0.030), at 6 weeks after BCG (28.0% *vs* 12.2%, *P* = 0.030), at 3 months after BCG (25.0% *vs* 6.8%, *P* = 0.013), and at 6 months after BCG (69.2% *vs* 16.9%, *P* < 0.001)		
IL‐2	Watanabe *et al*.^43^	20					Elevated IL‐2 levels are an independent predictor of BCG response (risk ratio 0.368, 95% CI 0.29–0.895, *P* = 0.003)
	de Reijke *et al*.^44^	23	Low levels of urinary IL‐2 levels correlated significantly with tumor recurrence within 6 months after BCG initiation (*P* = 0.003)				

Adamkiewicz *et al*. contextualized NLR, PLR, and LMR to identify the best marker of progression in bladder cancer patients receiving BCG – all markers independently prognosticated progression in multivariable analysis.^23^ However, when compared alongside one another, LMR was superior to NLR and PLR in terms of predicting disease progression, surpassing as the indicator with the strongest prognostic value.

Most of the studied biomarkers were serum in nature. However, Kamat *et al*.^31^ did evaluate the role of FISH in urine samples collected during BCG immunotherapy. FISH was performed using the UroVysion Bladder Cancer Recurrence Kit on patients who received the induction and maintenance course according to the Southwest Oncology Group trial 8507 protocol.^32^, ^33^ The study found that the presence of positive FISH results correlated with recurrence and progression rates. The same center also derived a cytokine panel based on nine urinary cytokines measured from before to just after the sixth instillation of BCG which predicted for recurrence with an AUC of 0.855 (95% CI 0.779–0.931).^34^

### Genomic pathway alterations

**Fig. 1:**
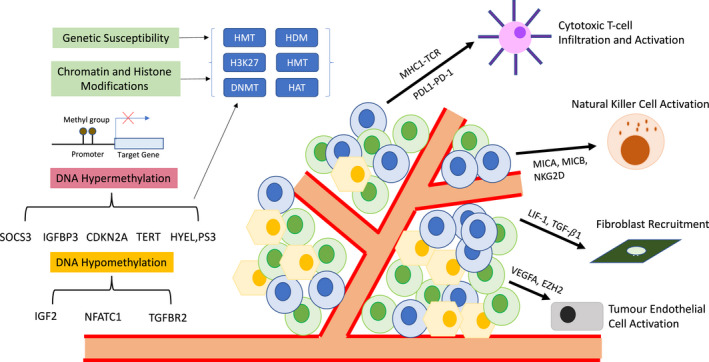
Epigenetic–genetic interactions in bladder cancer TME, providing differing mechanisms of control that regulates oncogene expression and silencing. [Colour figure can be viewed at wileyonlinelibrary.com]

In particular, the research in p53, pRB, and PD‐L1 expression in bladder cancer has gained considerable traction in the last few years.^35^, ^36^, ^37^, ^38^, ^39^, ^40^ Genomic pathway alterations can generally be categorized into SNPs, CNV alterations, and epigenetic‐related modulation of genes,^41^ as seen in Figure 1. One of the earlier established single‐nucleotide variances was related to the interleukin cytokine family, whereby Leibovici *et al*. demonstrated that a particular variant genotype (C/C) of IL‐6 correlated with an increased risk of disease recurrence (HR 4.60) in patients receiving maintenance BCG after TURBT.^42^ Other studies have also demonstrated that high urinary levels of IL‐2 after BCG instillation were associated with shorter recurrence‐free survival.^43^, ^44^ While variances of tumour necrosis factor α and IL‐8 were associated with a lower risk of disease recurrence and longer recurrence‐free survival in BCG‐treated TURBT patients,^45^ IL‐2, IL‐4, IL‐17, and monocyte chemoattractant protein 1 demonstrated significant correlation with disease recurrence after BCG therapy.^46^

The Sonic Hedgehog Pathway (Shh) is another crucial cell signaling pathway that controls tumor proliferation and differentiation. Chen *et al*.^47^ comparatively evaluated 177 SNPs found within 11 Shh pathways, reporting that nine SNPs located on GLI3, GLI2, and HHIP were all significantly associated with time to recurrence after BCG instillation (*P* < 0.05). Furthermore, two variant genotypes rs6463089 and rs3801192 remained significantly associated with a 2.40 (95% CI 1.50–3.84) and 2.54‐fold (95% CI 1.47–4.39) risk in recurrence after BCG treatment, respectively.

Copy number variations have been extensively studied in its relations to outcomes of bladder cancer, but there is a paucity of data on its role in predicting for BCG therapy success. Cai *et al*. explored the impact of loss of heterozygosity on the interferon‐α gene locus and its relation to BCG treatment outcomes.^48^ Survival curve analysis revealed a significant association between the risk of recurrence and loss of heterozygosity on interferon‐α (*P* < 0.0001). On multivariate analyses, loss of heterozygosity remained as an independent predictor of BCG response (HR 4.09; 95% CI 2.59–6.28; *P* = 0.002).

Agundez *et al*. assessed the role of 25 methylated TSGs and their association with clinical outcomes of BCG‐treated T1G3 tumors.^49^ The study evaluated several TSGs, notably PAX6, MSH6, RB1, THBS1, PYCARD, TP73, ESR1, and GATA. Two TSGs – MSH6 and THBS1 – were noted to be the predictors of disease progression after BCG on multivariate analyses.

In particular, the research in PD‐L1 expression in bladder cancer has gained considerable traction in the last few years. Of note, Pierconti *et al*.^50^ demonstrated a significant correlation of PD‐L1 with tumor recurrence (*P* = 0.035). BCG nonresponders also were observed to have elevated PD‐L1 levels compared to responders. The phase 2 study KEYNOTE‐057^51^ suggested that pembrolizumab was a possible nonsurgical option for patients with BCG unresponsive NMIBC who were not keen for or candidates for radical cytectomies. A complete response rate of 41% at 3 months and 23% for 2 years was achieved in BCG unresponsive bladder cancer patients with CIS who declined or were ineligible for radical cystectomy. Notably, complete response rates seemed to be better in patients with PDL‐1 negative status (combined positive score < 10%) patients. A summary of the genomic pathway alterations associated with outcomes of intravesical BCG therapy in NMIBC is outlined in Table 2.

**Table 2:** Genomic pathway alterations associated with the outcomes of intravesical BCG therapy in NMIBC

Predictors	Author	Number of patients/samples	Outcome				
			Recurrence (*P*‐value/HR/OR/correlation coefficient [*R*])	Recurrence‐free survival (*P*‐value/HR/OR/correlation coefficient [*R*])	Progression (*P*‐value/HR/OR/correlation coefficient [*R*])	Progression‐free survival (*P*‐value/HR/OR/correlation coefficient [*R*])	Response (*P*‐value/HR/OR/correlation coefficient [*R*])
IL‐6 variant genotype (C/C)	Leibovici *et al*.^42^	519	HR 4.60 (95% CI 1.24–17.09)		HR 1.88 (95% CI 0.80–4.11)		
IL‐2 receptor alpha rs2104286	Lima *et al*.^46^	204	aHR 2.007 (95% CI 1.207–3.335, *P* = 0.007)				
IL‐17 (IL‐17A rs2275913)	Lima *et al*.^46^	204	aHR 2.097 (95% CI 1.118–3.993, *P* = 0.021)				
TNF‐alpha T‐1031C (CC genotype)	Ahirwar *et al*.^45^	426	HR 0.38 (95% CI 0.14–0.98, *P* = 0.024)	Improved RFS at 31 and 60 months (*P* = 0.024)			
TNF‐alpha T‐1031C (CT genotype)	Ahirwar *et al*.^45^	426	HR 1.27 (95% CI 0.56–2.84)				
TNF‐alpha rs1799964	Lima *et al*.^46^	204	aHR 2.427 (95% CI 1.144–5.149, *P* = 0.021)				
Intercellular adhesion molecule 1 (ICAM‐1) rs5498	Lima *et al*.^46^	204	aHR 1.759 (95% CI 1.050–2.949, *P* = 0.032)				
C‐C chemokine receptor type 2 (CCR2 rs391835)	Lima *et al*.^46^	204	aHR 2.197 (95% CI 1.120–4.312, *P* = 0.022)				
Sonic Hedgehog (Shh) pathway SNP genetic variations	Chen *et al*.^47^	419	GLI3 rs6463089 HR 2.40 (95% CI 1.50–3.84) rs3801192 HR 2.54 (95% CI 1.47–4.39)				
Interferon‐alpha (chromosome 9p21)	Cai *et al*.^48^	117	HR 5.002 (95% CI 2.459–10.17, *P* < 0.0001)		HR 4.09 (95% CI 2.59–6.28, *P* = 0.002)		
p53	Esuvaranathan *et al*.^35^	80	p53 positive *vs* p53 negative (36.8% *vs* 32.4%, *P* = 0.741)		p53 positive *vs* p53 negative (15.8% *vs* 5.9%, *P* = 0.336)		
	Park *et al*.^36^	61		5‐year RFS: <10% p53‐positive cells *vs* ≥10% p53‐positive cells (49.9% *vs* 59.3%, *P* = 0.792)		5‐year PFS: <10% p53‐positive cells *vs* ≥10% p53‐positive cells (90.7% *vs* 78.7%, *P* = 0.0495)	
	Lee *et al*.^37^	32	RR 3.8 (95% CI 1.3–11.4, *P* = 0.0182)				
	Cormio *et al*.^38^	27	Altered p53 expression *vs* normal p53 expression (38% *vs* 36%, *P* = 0.92)		Altered p53 expression *vs* normal p53 expression (31% *vs* 0%, *P* = 0.06)		
	Gaya *et al*.^39^	134	p53 positive *vs* p53 negative (44.4% *vs* 46.8%, *P* = 0.83)				
	Lopez‐Beltran *et al*.^40^	51		≤2% p53‐positive cells *vs* >2% p53‐positive cells (60% *vs* 30.56%, *P* = 0.0332)		≤6% p53‐positive cells *vs* >6% p53‐positive cells RR 1.037 (95% CI 1.002–1.074, *P* = 0.039)	
pRB	Cormio *et al*.^38^	27	Altered pRB expression *vs* normal pRB expression (57% *vs* 15%, *P* = 0.037)		Altered pRB expression *vs* normal pRB expression (36% *vs* 0%, *P* = 0.018)		
	Park *et al*.^36^	61		5‐year RFS: 1–50% pRB‐positive cells *vs* 0%/>50% pRB‐positive cells (60.9% *vs* 53.6%, *P* = 0.951)		5‐year PFS: 1–50% pRB‐positive cells *vs* 0%/>50% pRB‐positive cells (87.1% *vs* 83.0%, *P* = 0.912)	
PD‐L1 expression	Pierconti *et al*.^50^	65	OR 0.1204 (95% CI 0.0147–1.023, *P* = 0.035)				

#### Tumor mutation burden

Bladder cancer is one of the cancers with the highest mutational burden, compared to other tumor types. TMB was first studied by Meeks *et al*., who found a significantly lower TMB in progressors compared to nonprogressors.^52^ This was also correlated by the Brazilian group^53^ in 2020. Bastos *et al*. performed whole exome sequencing from tumor samples extracted from 35 patients with NMIBC who were treated with TURBT, re‐TUR, and six or more BCG instillations. They also found that TMB load was higher in patients who were BCG responsive compared to those who were BCG relapsing or BCG refractory.

However, Meek's cohort group had a much higher TMB, ranging between 5 and 15 mutations/MB compared to the Bastos' group, which had a median TMB of 3 mutations/MB, with a range of 2.6–5.5 mutations/MB. In multivariate analyses, a high TMB was still an important biomarker of BCG response, with an OR of 5.20 (95% CI 1.11–24.34).

#### DNA methylation

DNA methylation has played an central role in epigenetic‐related gene modulation and was potentially recognized as an alternative molecular marker that could predict clinical outcomes of NMIBC patients treated with BCG, initially pioneered by Alvarez‐Múgica *et al*.’s single gene analysis.^54^ Subsequently, another genome‐wide methylation analysis was performed on a cohort of high‐risk NMIBC patients, concluding that the presence DNA methylation independently predicted 1‐year clinical outcomes.^55^

Ilijazi *et al*.^56^ found that gene promoter GPR158, when hypermethylated, was found to be the ideal predictor of BCG failure with an AUC value of 0.809 (*P* < 0.001). However, most of the methylation studies have modest sample sizes and varied definitions of threshold methylation differences, hence DNA methylation remains to be established as a reliable biomarker for BCG responsiveness.

#### Neoantigen load

Neoantigens are nonautologous, tumor‐specific antigens produced as a result of tumor gene mutation.^57^ NMIBC specimens from 35 patients who undergone BCG treatment were analyzed using whole exome sequencing and then classified as BCG responsive and unresponsive respectively.^53^ NAL was found to be greater in BCG responders (NAL: 100; IQR 75–145 *vs* NAL: 65; IQR 48.3–82.5; *P* = 0.032). Elevated NAL levels also significantly correlated with higher BCG response rates and lower recurrence rates, with a recurrence‐free survival of 76% *vs* 33% at 24 months. In multivariate analyses, NAL remained an independent predictor of BCG response with an OR of 6.57 (95% CI 1.32–32.67).

#### Inflamed TME

A constitutively inflamed environment has been acknowledged as an integral hallmark of bladder carcinogenesis.^58^ This prompted a study by Damrauer *et al*. to analyze untested gene expression signatures and establish possible associations with clinical and treatment endpoints.^59^ A RNA‐based profiling (NanoString nCounter) was conducted on samples of NMIBC and reported the presence of a novel molecular expression signature of an inflamed TME, which significantly correlated with longer recurrence‐free survival after BCG therapy. Conversely, the study also identified an immunologically “cold” variant of TME of which if present signified BCG unresponsiveness. Advanced transcriptomic and sequencing also uncovered associations between the “cold” TME and FGFR3 gene overexpression.

### Molecular subtypes

The concept of molecular subtyping was extrapolated from studies based on breast cancer. Sjodahl^60^ derived a Lund classification system based on distinct gene expression signatures specific for cell cycle, cytokeratins, cell adhesion, receptor tyrosine kinases, and immune response. The original urothelial carcinoma subtypes were defined as urobasal A, genomically unstable, urobasal B, squamous cell carcinoma like, and an infiltrated class of tumors.

However, this classification was mostly based on whole genome sequencing. The UROMOL multicenter collaboration utilized total RNA sequencing and paired‐end sequencing. The transcriptome sequencing enabled them to reclassify the tumors into three major classes, with basal‐ and luminal‐like features. The presence of stage Ta and T1 tumors with basal‐like characteristics (class 3) were hypothesized to represent a Ta pathway of disease progression, while the luminal‐like tumors (class 2) were hypothesized to follow the CIS pathway of progression. The authors were unable to detect any statistical differences to BCG response among the three groups, but it should be noted that a significant proportion of the patients with CIS did not receive any BCG.

This work was subsequently elaborated on by Robertson *et al*. who specifically evaluated the cohort of The Cancer Genome Atlas patients with high‐grade T1 NMIBC treated with BCG, with a primary outcome of interest deemed as recurrence after BCG instillation.^61^ The Bladder Cancer Molecular Taxonomy Group developed a five‐cluster solution with objectively delineated clinical endpoints and biological features for each subtype: T1‐LumGU, T1‐Inflam, T1‐Myc, T1‐TLum, and T1‐Early. T1‐Myc and T1‐Early molecular subtypes experienced the highest rate of recurrence after BCG (14/30 with 24 months) and collectively had significantly poorer recurrence‐free survival in comparison to the other three subtypes. They found that these prognostic subtypes corresponded with established subtype methods – T1‐LumGU demonstrated comparable findings to LumU subtype in MIBC,^62^ while T1‐Inflam demonstrated similarity to the class 2b subtype in the UROMOL classification system^63^ as well as the basal and mesenchymal‐like subtypes in the Lund classification. Kim *et al*. also suggested another subtyping system to predict response to BCG therapy. Within their cohort of 948 NMIBC patients, they reported a prognostic signature‐based subtype after transcriptional profiling^64^ and found that MSP888 was able to independently predict both NMIBC progression and recurrence after BCG and was largely on‐par with the Lund taxonomy and UROMOL system in terms of subtype homogeneity across all classifications. The other predictors associated with the outcomes of intravesical BCG therapy in NMIBC are delineated in Table 3.

**Table 3:** Other predictors associated with the outcomes of intravesical BCG therapy in NMIBC

Predictors	Author	Number of patients/samples	Outcome				
			Recurrence (*P*‐value/HR/OR/correlation coefficient [*R*])	Recurrence‐free survival (*P*‐value/HR/OR/correlation coefficient [*R*])	Progression (*P*‐value/HR/OR/correlation coefficient [*R*])	Progression‐free survival (*P*‐value/HR/OR/correlation coefficient [*R*])	Response (*P*‐value/HR/OR/correlation coefficient [*R*])
Tumor mutation burden	Bastos *et al*.^53^	35	HR 0.28 (95% CI 0.10–0.82, *P* = 0.021)	HR 0.27 (95% CI 0.09–0.77, *P* = 0.009)			OR 5.20 (95% CI 1.11–24.34, *P* = 0.036)
DNA methylation	Kitchen *et al*.^55^	21	Hypermethylation of CpG cg11850659 and hypomethylation of CpG cg01149192 predicted recurrence and progression within 1‐year diagnosis of NMIBC (83% sensitivity, 79% specificity, 83% positive predictive value, and 79% negative predictive value)				
	Illijazi *et al*.^56^	53	GPR158 promoter hypermethylation was a predictor of BCG failure (AUC 0.809, *P* < 0.001)				
NAL	Bastos *et al*.^53^	35		HR 0.28 (95% CI 0.10–0.81, *P* = 0.019)			OR 6.57 (95% CI 1.32–32.67)
MSP888 molecular signature‐based subtype	Kim *et al*.^64^	948		HR 2.569 (95% CI 1.065–6.915, *P* = 0.036)		HR 3.447 (95% CI 1.483–8.012, *P* = 0.004)	

## Conclusion

The group of patients who are BCG unresponsive remains a heterogeneous cohort. The above studies have laid the initial foundation to permit stratification on many factors but are largely still hypothesis generating in nature. Future translational studies should be performed in more ethnically diverse populations to elucidate the functional significance of the biomarkers in the management of BCG unresponsive bladder cancer and access potential therapeutic options.

## Author contributions

Ziting Wang: Conceptualization; data curation; formal analysis; investigation; methodology; writing – original draft. Wei Zheng So: Conceptualization; data curation; writing – original draft; writing – review and editing. Kep Yong Loh: Conceptualization; data curation; software; visualization. Yew Koon Lim: Conceptualization; data curation. Ratha Mahendran: Writing – review and editing. Qing Hui Wu: Writing – review and editing. Edmund Chiong: Conceptualization; data curation; formal analysis; investigation; writing – review and editing.

## Conflict of interest

None declared.

## References

[1] Babjuk M , Burger M , Capoun O *et al*. European Association of Urology Guidelines on non‐muscle‐invasive bladder cancer (Ta, T1, and carcinoma in situ). Eur. Urol. 2021; 1: 31. 10.1016/j.eururo.2021.08.010 34511303

[2] Kamat AM , Bellmunt J , Galsky MD *et al*. Society for Immunotherapy of Cancer consensus statement on immunotherapy for the treatment of bladder carcinoma. J. Immunother. Cancer 2017; 5: 68. 28807024 10.1186/s40425-017-0271-0 PMC5557323

[3] Kamat AM , Flaig TW , Grossman HB *et al*. Consensus statement on best practice management regarding the use of intravesical immunotherapy with BCG for bladder cancer. Nat. Rev. Urol. 2015; 12: 225–35. 25800393 10.1038/nrurol.2015.58

[4] Babjuk M , Oosterlinck W , Sylvester R , Kaasinen E , Böhle A , Palou‐Redorta J . EAU guidelines on non‐muscle‐invasive urothelial carcinoma of the bladder. Eur. Urol. 2008; 54: 303–14. 18468779 10.1016/j.eururo.2008.04.051

[5] Sylvester RJ , Van Der Meijden AP , Oosterlinck W *et al*. Predicting recurrence and progression in individual patients with stage Ta T1 bladder cancer using EORTC risk tables: a combined analysis of 2596 patients from seven EORTC trials. Eur. Urol. 2006; 49: 466–77. 16442208 10.1016/j.eururo.2005.12.031

[6] Babjuk M , Oosterlinck W , Sylvester R *et al*. EAU guidelines on non‐muscle‐invasive urothelial carcinoma of the bladder, the 2011 update. Eur. Urol. 2011; 59: 997–1008. 21458150 10.1016/j.eururo.2011.03.017

[7] Babjuk M , Burger M , Zigeuner R *et al*. EAU guidelines on non–muscle‐invasive urothelial carcinoma of the bladder: update 2013. Eur. Urol. 2013; 64: 639–53. 23827737 10.1016/j.eururo.2013.06.003

[8] Babjuk M , Böhle A , Burger M *et al*. EAU guidelines on non–muscle‐invasive urothelial carcinoma of the bladder: update 2016. Eur. Urol. 2017; 71: 447–61. 27324428 10.1016/j.eururo.2016.05.041

[9] Babjuk M , Burger M , Compérat EM *et al*. European Association of Urology guidelines on non‐muscle‐invasive bladder cancer (TaT1 and carcinoma in situ) – 2019 update. Eur. Urol. 2019; 76: 639–57. 31443960 10.1016/j.eururo.2019.08.016

[10] Krajewski W , Rodríguez‐Faba O , Breda A *et al*. Validation of the CUETO scoring model for predicting recurrence and progression in T1G3 urothelial carcinoma of the bladder. Actas Urol. Esp. 2019; 43: 445–51. 31155372 10.1016/j.acuro.2019.02.006

[11] Xylinas E , Kent M , Kluth L *et al*. Accuracy of the EORTC risk tables and of the CUETO scoring model to predict outcomes in non‐muscle‐invasive urothelial carcinoma of the bladder. Br. J. Cancer 2013; 109: 1460–6. 23982601 10.1038/bjc.2013.372 PMC3776972

[12] Hurle R , Losa A , Manzetti A , Lembo A . Intravesical bacille Calmette‐Guerin in stage T1 grade 3 bladder cancer therapy: a 7‐year follow‐up. Urology 1999; 54: 258–63. 10443721 10.1016/s0090-4295(99)00116-8

[13] Fernandez‐Gomez J , Solsona E , Unda M *et al*. Prognostic factors in patients with non–muscle‐invasive bladder cancer treated with bacillus Calmette‐Guérin: multivariate analysis of data from four randomized CUETO trials. Eur. Urol. 2008; 53: 992–1002. 17950987 10.1016/j.eururo.2007.10.006

[14] Gontero P , Sylvester R , Pisano F *et al*. Prognostic factors and risk groups in T1G3 non–muscle‐invasive bladder cancer patients initially treated with bacillus Calmette‐Guérin: results of a retrospective multicenter study of 2451 patients. Eur. Urol. 2015; 67: 74–82. 25043942 10.1016/j.eururo.2014.06.040

[15] Fernandez‐Gomez J , Madero R , Solsona E *et al*. Predicting nonmuscle invasive bladder cancer recurrence and progression in patients treated with bacillus Calmette‐Guerin: the CUETO scoring model. J. Urol. 2009; 182: 2195–203. 19758621 10.1016/j.juro.2009.07.016

[16] Mungan NA , Kiemeney LA , van Dijck JA , van der Poel HG , Witjes JA . Gender differences in stage distribution of bladder cancer. Urology 2000; 55: 368–71. 10699612 10.1016/s0090-4295(99)00481-1

[17] Tuygun C , Kankaya D , Imamoglu A *et al*. Sex‐specific hormone receptors in urothelial carcinomas of the human urinary bladder: a comparative analysis of clinicopathological features and survival outcomes according to receptor expression. Urol. Oncol.: Semin. Orig. Investig. 2011; 29: 43–51. 10.1016/j.urolonc.2009.01.033 19372057

[18] Miyake M , Tatsumi Y , Gotoh D *et al*. Regulatory T cells and tumor‐associated macrophages in the tumor microenvironment in non‐muscle invasive bladder cancer treated with intravesical Bacille Calmette‐Guérin: a long‐term follow‐up study of a Japanese cohort. Int. J. Mol. Sci. 2017; 18: 2186. 10.3390/ijms18102186 PMC5666867 29048388

[19] Herr HW . Age and outcome of superficial bladder cancer treated with Bacille Calmette‐Guérin therapy. Urology 2007; 70: 65–8. 17656210 10.1016/j.urology.2007.03.024

[20] Oddens JR , Sylvester RJ , Brausi MA *et al*. The effect of age on the efficacy of maintenance bacillus Calmette‐Guérin relative to maintenance epirubicin in patients with stage ta T1 urothelial bladder cancer: results from EORTC genito‐urinary group study 30911. Eur. Urol. 2014; 66: 694–701. 24948466 10.1016/j.eururo.2014.05.033

[21] Fuentes E , Fuentes M , Alarcon M , Palomo I . Immune system dysfunction in the elderly. An. Acad. Bras. Cienc. 2017; 89: 285–99. 28423084 10.1590/0001-3765201720160487

[22] Saint F , Patard JJ , Irani J *et al*. Leukocyturia as a predictor of tolerance and efficacy of intravesical BCG maintenance therapy for superficial bladder cancer. Urology 2001; 57: 617–21. 11306359 10.1016/s0090-4295(01)00921-9

[23] Adamkiewicz M , Bryniarski P , Kowalik M , Burzyński B , Rajwa P , Paradysz A . Lymphocyte‐to‐monocyte ratio is the independent prognostic marker of progression in patients undergoing BCG‐immunotherapy for bladder cancer. Front. Oncol. 2021; 11: 655000. 33842371 10.3389/fonc.2021.655000 PMC8033152

[24] Lee S‐M , Russell A , Hellawell G . Predictive value of pretreatment inflammation‐based prognostic scores (neutrophil‐to‐lymphocyte ratio, platelet‐to‐lymphocyte ratio, and lymphocyte‐to‐monocyte ratio) for invasive bladder carcinoma. Korean J. Urol. 2015; 56: 749–55. 26568792 10.4111/kju.2015.56.11.749 PMC4643170

[25] Ma J‐y , Hu G , Liu Q . Prognostic significance of the lymphocyte‐to‐monocyte ratio in bladder cancer undergoing radical cystectomy: a meta‐analysis of 5638 individuals. Dis. Markers 2019; 2019: 7593560. 31089397 10.1155/2019/7593560 PMC6476040

[26] Vartolomei MD , Ferro M , Cantiello F *et al*. Validation of neutrophil‐to‐lymphocyte ratio in a multi‐institutional cohort of patients with T1G3 non–muscle‐invasive bladder cancer. Clin. Genitourin. Cancer 2018; 16: 445–52. 30077463 10.1016/j.clgc.2018.07.003

[27] Racioppi M , Di Gianfrancesco L , Ragonese M , Palermo G , Sacco E , Bassi PF . Can neutrophil‐to‐lymphocyte ratio predict the response to BCG in high‐risk non muscle invasive bladder cancer? Int. Braz. J. Urol. 2019; 45: 315–24. 30785697 10.1590/S1677-5538.IBJU.2018.0249 PMC6541147

[28] Getzler I , Bahouth Z , Nativ O , Rubinstein J , Halachmi S . Preoperative neutrophil to lymphocyte ratio improves recurrence prediction of non‐muscle invasive bladder cancer. BMC Urol. 2018; 18: 90. 30348146 10.1186/s12894-018-0404-x PMC6198354

[29] Bao Y , Wang Y , Li X *et al*. Prognostic significance of platelet‐to‐lymphocyte ratio in urothelial carcinoma patients: a meta‐analysis. Cancer Cell Int. 2019; 19: 315. 31798344 10.1186/s12935-019-1032-6 PMC6882352

[30] Bi H , Yan Y , Wang D *et al*. Predictive value of preoperative lymphocyte‐to‐monocyte ratio on survival outcomes in bladder cancer patients after radical cystectomy. J. Cancer 2021; 12: 305. 33391427 10.7150/jca.50603 PMC7738993

[31] Kamat AM , Dickstein RJ , Messetti F *et al*. Use of fluorescence in situ hybridization to predict patient response to BCG therapy for bladder cancer: results of a prospective trial. J. Urol. 2012; 187: 862. 22245325 10.1016/j.juro.2011.10.144 PMC3278506

[32] Lamm D . Maintenance versus no‐maintenance BCG‐immunotherapy of superficial bladder cancer. J. Urol. 1992; 147: 274.1729545

[33] Lamm DL , Blumenstein BA , Crissman JD *et al*. Maintenance bacillus Calmette‐Guerin immunotherapy for recurrent TA, T1 and carcinoma in situ transitional cell carcinoma of the bladder: a randomized southwest oncology group study. J. Urol. 2000; 163: 1124–9. 10737480

[34] Kamat AM , Briggman J , Urbauer DL *et al*. Cytokine panel for response to intravesical therapy (CyPRIT): nomogram of changes in urinary cytokine levels predicts patient response to bacillus Calmette‐Guérin. Eur. Urol. 2016; 69: 197–200. 26119560 10.1016/j.eururo.2015.06.023 PMC4691211

[35] Esuvaranathan K , Chiong E , Thamboo TP *et al*. Predictive value of p53 and pRb expression in superficial bladder cancer patients treated with BCG and interferon‐alpha. Cancer 2007; 109: 1097–105. 17311305 10.1002/cncr.22503

[36] Park J , Song C , Shin E , Hong JH , Kim C‐S , Ahn H . Do molecular biomarkers have prognostic value in primary T1G3 bladder cancer treated with bacillus Calmette‐Guerin intravesical therapy? Urol. Oncol. 2013; 31: 849–56. 21782482 10.1016/j.urolonc.2011.06.004

[37] Lee E , Park I , Lee C . Prognostic markers of intravesical bacillus Calmette‐Guerin therapy for multiple, high‐grade, stage T1 bladder cancers. Int. J. Urol. 1997; 4: 552–6. 9477182 10.1111/j.1442-2042.1997.tb00307.x

[38] Cormio L , Tolve I , Annese P *et al*. Altered p53 and pRb expression is predictive of response to BCG treatment in T1G3 bladder cancer. Anticancer Res. 2009; 29: 4201–4. 19846973

[39] Gaya JM , López‐Martínez JM , Karni‐Schmidt O *et al*. ΔNp63 expression is a protective factor of progression in clinical high grade T1 bladder cancer. J. Urol. 2015; 193: 1144–50. 25444981 10.1016/j.juro.2014.10.098

[40] Lopez‐Beltran A , Luque R , Alvarez‐Kindelan J *et al*. Prognostic factors in stage T1 grade 3 bladder cancer survival: the role of G1–S modulators (p53, p21Waf1, p27Kip1, cyclin D1, and cyclin D3) and proliferation index (ki67‐MIB1). Eur. Urol. 2004; 45: 606–12. 15082203 10.1016/j.eururo.2003.11.011

[41] Zhang N , Jiang G , Liu X , Na R , Wang X , Xu J . Prediction of bacillus Calmette‐Guerin response in patients with bladder cancer after transurethral resection of bladder tumor by using genetic variation based on genomic studies. Biomed. Res. Int. 2016; 2016: 9859021. 27896277 10.1155/2016/9859021 PMC5118509

[42] Leibovici D , Grossman HB , Dinney CP *et al*. Polymorphisms in inflammation genes and bladder cancer: from initiation to recurrence, progression, and survival. J. Clin. Oncol. 2005; 23: 5746–56. 16110031 10.1200/JCO.2005.01.598

[43] Watanabe E , Matsuyama H , Matsuda K *et al*. Urinary interleukin‐2 may predict clinical outcome of intravesical bacillus Calmette‐Guerin immunotherapy for carcinoma in situ of the bladder. Cancer Immunol. Immunother. 2003; 52: 481–6. 12707736 10.1007/s00262-003-0384-9 PMC11032925

[44] de Reijke TM , de Boer EC , Kurth KH , Schamhart DH . Urinary cytokines during intravesical bacillus Calmette‐Guerin therapy for superficial bladder cancer: processing, stability and prognostic value. J. Urol. 1996; 155: 477–82. 8558640

[45] Ahirwar DK , Mandhani A , Dharaskar A , Kesarwani P , Mittal RD . Association of tumour necrosis factor‐α gene (T‐1031C, C‐863A, and C‐857T) polymorphisms with bladder cancer susceptibility and outcome after bacille Calmette‐Guérin immunotherapy. BJU Int. 2009; 104: 867–73. 19338536 10.1111/j.1464-410X.2009.08549.x

[46] Lima L , Oliveira D , Ferreira JA *et al*. The role of functional polymorphisms in immune response genes as biomarkers of bacille Calmette‐Guérin (BCG) immunotherapy outcome in bladder cancer: establishment of a predictive profile in a southern Europe population. BJU Int. 2015; 116: 753–63. 24931268 10.1111/bju.12844

[47] Chen M , Hildebrandt MA , Clague J *et al*. Genetic variations in the sonic hedgehog pathway affect clinical outcomes in non‐muscle‐invasive bladder cancer. Cancer Prev. Res. 2010; 3: 1235–45. 10.1158/1940-6207.CAPR-10-0035 PMC2955764 20858759

[48] Cai T , Nesi G , Dal Canto M *et al*. Loss of heterozygosis on IFN‐alpha locus is a prognostic indicator of bacillus Calmette‐Guerin response for nonmuscle invasive bladder cancer. J. Urol. 2010; 183: 1738–43. 20299058 10.1016/j.juro.2009.12.105

[49] Agundez M , Grau L , Palou J , Algaba F , Villavicencio H , Sanchez‐Carbayo M . Evaluation of the methylation status of tumour suppressor genes for predicting bacillus Calmette‐Guérin response in patients with T1G3 high‐risk bladder tumours. Eur. Urol. 2011; 60: 131–40. 21514719 10.1016/j.eururo.2011.04.020

[50] Pierconti F , Raspollini MR , Martini M *et al*. PD‐L1 expression in bladder primary in situ urothelial carcinoma: evaluation in BCG‐unresponsive patients and BCG responders. Virchows Arch. 2020; 477: 269–77. 32034486 10.1007/s00428-020-02755-2

[51] Balar AV , Kamat AM , Kulkarni GS *et al*. Pembrolizumab monotherapy for the treatment of high‐risk non‐muscle‐invasive bladder cancer unresponsive to BCG (KEYNOTE‐057): an open‐label, single‐arm, multicentre, phase 2 study. Lancet Oncol. 2021; 22: 919–30. 34051177 10.1016/S1470-2045(21)00147-9

[52] Meeks JJ , Carneiro BA , Pai SG *et al*. Genomic characterization of high‐risk non‐muscle invasive bladder cancer. Oncotarget 2016; 7: 75176. 27750214 10.18632/oncotarget.12661 PMC5342732

[53] Bastos DA , Mattedi RL , Barreiro R *et al*. Genomic biomarkers and underlying mechanism of benefit from BCG immunotherapy in non‐muscle invasive bladder cancer. Bladder Cancer 2020; 6: 171–86.

[54] Alvarez‐Múgica M , Fernández‐Gómez JM , Cebrian V , Fresno F , Escaf S , Sánchez‐Carbayo M . Polyamine‐modulated factor‐1 methylation predicts bacillus Calmette‐Guérin response in patients with high‐grade non‐muscle‐invasive bladder carcinoma. Eur. Urol. 2013; 63: 364–70. 22682992 10.1016/j.eururo.2012.05.050

[55] Kitchen MO , Bryan RT , Emes RD *et al*. HumanMethylation450K Array‐identified biomarkers predict tumour recurrence/progression at initial diagnosis of high‐risk non‐muscle invasive bladder cancer. Biomark Cancer 2018; 10: 1179299x17751920. 10.1177/1179299X17751920 PMC5764140 29343995

[56] Ilijazi D , Pulverer W , Ertl IE *et al*. Discovery of molecular DNA methylation‐based biomarkers through genome‐wide analysis of response patterns to BCG for bladder cancer. Cell 2020; 9: 1839. 10.3390/cells9081839 PMC7464079 32764425

[57] Wang P , Chen Y , Wang C . Beyond tumor mutation burden: tumor neoantigen burden as a biomarker for immunotherapy and other types of therapy. Front. Oncol. 2021; 11: 672677. 33996601 10.3389/fonc.2021.672677 PMC8117238

[58] Sui X , Lei L , Chen L , Xie T , Li X . Inflammatory microenvironment in the initiation and progression of bladder cancer. Oncotarget 2017; 8: 93279. 29190997 10.18632/oncotarget.21565 PMC5696263

[59] Damrauer JS , Roell KR , Smith MA *et al*. Identification of a novel inflamed tumor microenvironment signature as a predictive biomarker of bacillus Calmette‐Guerin immunotherapy in non‐muscle invasive bladder cancer. Clin. Cancer Res. 2021; 27: 4599–609. 34117034 10.1158/1078-0432.CCR-21-0205 PMC8416390

[60] Sjödahl G , Lauss M , Lövgren K *et al*. A molecular taxonomy for urothelial carcinoma. Clin. Cancer Res. 2012; 18: 3377–86. 22553347 10.1158/1078-0432.CCR-12-0077-T

[61] Robertson AG , Groeneveld CS , Jordan B *et al*. Identification of differential tumor subtypes of T1 bladder cancer. Eur. Urol. 2020; 78: 533–7. 32684305 10.1016/j.eururo.2020.06.048

[62] Kamoun A , de Reyniès A , Allory Y *et al*. A consensus molecular classification of muscle‐invasive bladder cancer. Eur. Urol. 2020; 77: 420–33. 31563503 10.1016/j.eururo.2019.09.006 PMC7690647

[63] Lindskrog SV , Prip FF , Lamy P *et al*. An integrated multi‐omics analysis identifies clinically relevant molecular subtypes of non‐muscle‐invasive bladder cancer. medRxiv 2020; 12: 2301. 10.1038/s41467-021-22465-w PMC8052448 33863885

[64] Kim SK , Park SH , Kim YU *et al*. A molecular signature determines the prognostic and therapeutic subtype of non‐muscle‐invasive bladder cancer responsive to intravesical bacillus Calmette‐Guérin therapy. Int. J. Mol. Sci. 2021; 22: 1450. 33535616 10.3390/ijms22031450 PMC7867154

